# A cycloruthenated complex, ruthenium (II) Z (RuZ) overcomes *in vitro* and *in vivo* multidrug resistance in cancer cells: A pivotal breakthrough

**DOI:** 10.2478/jtim-2023-0081

**Published:** 2023-07-05

**Authors:** Luqi Cao, Yuhao Xie, Zhesheng Chen, Charles R. Ashby

**Affiliations:** Institute for Biotechnology, St. John’s University, Queens, New York 11439, USA; Department of Pharmaceutical Sciences, College of Pharmacy and Health Sciences, St. John’s University, Queens, New York 11439, USA

Multidrug resistance (MDR) is one of the major obstacles that attenuates or abrogates the efficacy of the treatment of cancer.^[1]^ Previously, numerous studies have reported that certain metallo-complexed anticancer drugs, such as platinum (II)-based compounds, are efficacious in treating specific types of cancer.^[2]^ However, these compounds are not efficacious in MDR cancers. Consequently, there is a need for the synthesis and development of safe and efficacious metal-complexed anticancer drugs.

A study published in the journal, *Advanced Materials*, by Li *et al*.^[3]^ reported the synthesis and characterization of a self-assembled cyclometalated Ru (II) complex. Subsequent characterization of the physical properties of RuZ indicated that RuZ had a large molecular size at high concentrations and single RuZ complexes spontaneously assembled into nano-aggregates in aqueous media. RuZ has a high drug loading rate, and this, in combination with its typical octahedral structure, decreases its interaction with the ABCG2 and ABCB1 transporters, producing an increase in RuZ retention in drug-resistant cancer cells.

First, Li *et al*.^[3]^ determined the cytotoxicity of RuZ in 35 cancer cell lines. Their results indicated that RuZ significantly decreased the proliferation of cancer cell lines and was 2–60 times more potent than cisplatin. RuZ was efficacious in cancer cells that were resistant to cisplatin (PT), arsenic trioxide (As_2_O_3_), doxorubicin (Dox), and mitoxantrone (MX). The uptake of RuZ in MDA-MB-231/ADR breast cancer cells, as determined by inductively coupled plasma mass spectrometry (ICPMS), produced nano-aggregates of RuZ, which could be internalized into the cancer cells by endocytosis, due to the capacity of nanomaterials to deliver low molecular weight compounds to the cells.^[4]^

The overexpression of ABCB1 and ABCG2 transporters increases the likelihood of MDR and subsequently, therapeutic failure.^[4]^ Therefore, the interaction between RuZ and the ABCG2 and ABCB1 transporters was determined using molecular docking to obtain Autodock scores for these transporters. As previously reported, the anticancer drug. doxorubicin, a substrate for ABCG2 and ABCB1.^[5]^ Autodock scores of -9.37 and -9.01 kcal/mol respectively. In contrast, the RuZ Autodock scores for ABCG2 and ABCB1 were -4.88 and -5.88 kcal/mol, respectively, indicating that RuZ has a significantly lower affinity for these transporters.^[6]^ Therefore, it is unlikely that RuZ will be removed from the intracellular milieu of MDA-MB-231 cancer cells expressing the ABCG2 and ABCB1 transporters, *i.e*., there will not be a significant decrease in the levels of RuZ, thus, increasing the efficacy of RuZ. Next, experiments were conducted to determine the mechanisms that produce a decrease in MDA-MB-231/Adr cancer cells. The incubation of MDA-MB-231/Adr with 1.5 or 3 μmol/L of RuZ significantly increased the levels of reactive oxygen species (ROS), as indicated by a significant increase in the levels of OH radicals formed from the

interaction of RuZ with HO. Furthermore, the RuZ-_22_induced increase in ROS levels and magnitude of cell death were significantly decreased by N-acetylcysteine, a scavenger of ROS. These results suggested that RuZ can decrease cancer growth by increasing the levels of OH radicals. *In vitro*, in MDA-MB-231 cancer cells, RuZ significantly decreased the mitochondrial membrane potential and inhibited mitochondrial aerobic respiration, which decreased ATP synthesis. The incubation of MDAMB-231/Adr cancer cells with 1.5 or 3 μmol/L of RuZ produced DNA damage, indicating that RuZ can penetrate the nucleus. RuZ was shown to accumulate in the nucleus, mitochondria and cytoplasm of MDA-MB-231/Adr, as well as normal cells (LO2 human liver cells) and MCF10A (mammary epithelial cells). Using proteomics, it was shown that RuZ significantly decreased certain proteins involved in mitochondrial respiration and glycolysis (*e.g*., GAPDH, pyruvate kinase M2), producing a decrease in the levels of ATP and glucose, which decreased the efflux efficacy of ABCG2 and ABCB1 and decreased cancer cell viability, respectively. In contrast, RuZ significantly increased the levels of certain proteins that mediate cellular apoptosis, such as certain caspases. The peritumoral injection of 1.5 and 3 mg/ kg of RuZ in mice xenografted with MDA-MB-231/ Adr cancer cells produced a maximal inhibition of 33% and 62%, respectively, in the volume of the tumors, compared to mice treated with vehicle (control group). In addition, the peritumoral injection of 5 mg/ kg of doxorubicin for 2 weeks produced a significantly lower decrease in tumor volume, compared to 1.5 and 3 mg/ kg of RuZ. In mice, the peritumoral injection of 1.5 or 3 mg/kg of RuZ for 2 weeks did not significantly (1) affect the morphology of the heart, liver, spleen, lungs, kidneys, and intestines, and (2) body weight. These results suggested that RuZ did not produce significant *in vivo* toxicity, based on the doses, route of administration and length of treatment used in this study.

In conclusion, RuZ produced (1) an efficacious concentration in cancer cells; (2) an increase in ROS levels; (3) an increase in DNA damage; (4) a decrease in the levels of ATP and glucose, due to a decrease in the levels of proteins that regulate the levels of these biomolecules; (5) an increase in the levels of certain apoptotic proteins; (6) a significant decrease in the volume of xenografted MDAMD-231 tumors in mice; and (7) decreased the proliferation of 7 different MDR cancer cells. Furthermore, RuZ had a low affinity for ABCG2 and ABCB1, thus, indicating that RuZ is not extruded from MDA-MB-231 cancer cells. In mice with MDA-MB-231 tumors, RuZ, at the doses and length of treatment used in this study, did not produce any significant overt or organ toxicity.

Given that RuZ interacts with a number of cellular targets to produce cancer cell death, we hypothesized that the cancer cells would have a lower likelihood of becoming resistant to RuZ, as its efficacy is not dependent on its interaction with a single target. Indeed, after the incubation (for 48 hours) of various cancer cells (H23, H460, SW620, COLO-205, SF-539, SK-MEL-28, T-47D, MDA-MB-231 and HeLa) with 100 μmol/L of RuZ, there was only a 2-fold increase in the concentration required to decrease cell viability by 50%. This contrasts with certain anti-cancer drugs that produce a significant increase in their IC50 values following incubation with cancer cells. In the discovery and development of RuZ, Li *et al*.^[3]^ provided insights from multiple perspectives: analyzing the physical properties and chemical molecular structure of RuZ, determining the cytotoxicity of RuZ in 35 cancer cell lines (including MDR cell lines), determining the cellular uptake mechanism of RuZ, calculating the interaction between RuZ and the ABCG2 and ABCB1 transporters (which can mediate MDR) and determining the mechanisms by which RuZ decreases cancer cell viability. The efficacy of RuZ was also determined and compared to standard anticancer drugs. Experiments were conducted in mice to determine the biodistribution, toxicity and metabolism of RuZ, to provide guidance for the next steps in the development of RuZ as an anticancer drug. In order to determine the optimal dose range and adverse effects of RuZ, a self-assembling cyclic metallo-complex that may eradicate MDR cancer cells, will require additional *in vivo* experiments. Finally, the appropriate clinical trials must be conducted to assess the efficacy and safety of RuZ in cancer patients. It is possible that RuZ could be a treatment for certain types of MDR cancers.
